# Risk stratification of early admission to the intensive care unit of patients with no major criteria of severe community-acquired pneumonia: development of an international prediction rule

**DOI:** 10.1186/cc7781

**Published:** 2009-04-09

**Authors:** Bertrand Renaud, José Labarère, Eva Coma, Aline Santin, Jan Hayon, Mercé Gurgui, Nicolas Camus, Eric Roupie, François Hémery, Jérôme Hervé, Mirna Salloum, Michael J Fine, Christian Brun-Buisson

**Affiliations:** 1 Department of Emergency Medicine, AP-HP, Groupe Hospitalier Henri Mondor-Albert Chenevier, Créteil, F-94010, France; 2 Unité d'évaluation médicale, Centre Hospitalier Universitaire de Grenoble, Grenoble, F-38043, France; 3 Servei d'Atenció Continuada USAC, Institut Català d'Oncologia, Hospital Duran i Reynals, 08907 L'Hospitalet de Llobregat, Barcelona, Spain; 4 Department of Intensive Care Medicine, Centre Hospitalier Intercommunal de Poissy Saint-Germain, Saint-Germain-en-Laye, F-78100, France; 5 Department of Emergency Medicine, Hospital de la Santa Creu I Sant Pau, Barcelona, Spain; 6 Université Paris 12, Faculté de Médecine, Créteil, F-94000, France; 7 Department of Emergency Medicine, CHU de Caen, Hôpital Côte de Nacre, F-14033, Caen, France; 8 Université de Caen-Basse Normandie, Faculté de médecine, F-14032, Caen, France; 9 Département d'Informatique Hospitalier (PMSI et Recherche Clinique), AP-HP, Groupe Hospitalier Henri Mondor-Albert Chenevier, Créteil, F-94010, France; 10 Center for Health Equity Research and Promotion, VA Pittsburgh Healthcare System, 7180 Highland Drive (151C-H), Pittsburgh, PA 15206-1206, USA; 11 Division of General Internal Medicine, Department of Medicine, University of Pittsburgh, UPMC Montefiore Hospital, Suite W933, 200 Lothrop Street, Pittsburgh, PA 15213, USA; 12 AP-HP, Groupe hospitalier Henri Mondor-Albert Chenevier, Réanimation Médicale, Créteil, F-94010, France

## Abstract

**Introduction:**

To identify risk factors for early (< three days) intensive care unit (ICU) admission of patients hospitalised with community-acquired pneumonia (CAP) and not requiring immediate ICU admission, and to stratify the risk of ICU admission on days 1 to 3.

**Methods:**

Using the original data from four North American and European prospective multicentre cohort studies of patients with CAP, we derived and validated a prediction rule for ICU admission on days 1 to 3 of emergency department (ED) presentation, for patients presenting with no obvious reason for immediate ICU admission (not requiring immediate respiratory or circulatory support).

**Results:**

A total of 6560 patients were included (4593 and 1967 in the derivation and validation cohort, respectively), 303 (4.6%) of whom were admitted to an ICU on days 1 to 3. The Risk of Early Admission to ICU index (REA-ICU index) comprised 11 criteria independently associated with ICU admission: male gender, age younger than 80 years, comorbid conditions, respiratory rate of 30 breaths/minute or higher, heart rate of 125 beats/minute or higher, multilobar infiltrate or pleural effusion, white blood cell count less than 3 or 20 G/L or above, hypoxaemia (oxygen saturation < 90% or arterial partial pressure of oxygen (PaO_2_) < 60 mmHg), blood urea nitrogen of 11 mmol/L or higher, pH less than 7.35 and sodium less than 130 mEq/L. The REA-ICU index stratified patients into four risk classes with a risk of ICU admission on days 1 to 3 ranging from 0.7 to 31%. The area under the curve was 0.81 (95% confidence interval (CI) = 0.78 to 0.83) in the overall population.

**Conclusions:**

The REA-ICU index accurately stratifies the risk of ICU admission on days 1 to 3 for patients presenting to the ED with CAP and no obvious indication for immediate ICU admission and therefore may assist orientation decisions.

## Introduction

Approximately 10% of patients hospitalised for community-acquired pneumonia (CAP) are admitted to an intensive care unit (ICU), and these patients account for about 10% of all medical admissions to ICUs [1,2]. Although some patients with CAP have an obvious reason for ICU admission on the day of presentation to the emergency department (ED), a substantial proportion of others will develop organ failure within a few days [3]. Transfer to the ICU for delayed respiratory failure or delayed onset of septic shock is associated with increased mortality [4]. Hence, a major challenge in the management of CAP is to identify patients at risk for rapidly developing adverse medical outcomes among those presenting to the ED with no obvious reason for immediate ICU admission.

Since the publication of the American Thoracic Society (ATS) guidelines in 1993, several prediction rules have been derived to identify ED patients with severe CAP, defined by adverse outcomes (including ICU admission, shock requiring vasopressors, acute respiratory failure requiring mechanical ventilation or death). Most of these prediction rules were derived in populations including patients presenting with an obvious reason for immediate ICU admission. However, a prediction rule is essentially relevant to help management decisions for patients not requiring immediate respiratory or circulatory support at presentation to the ED [5]. Additionally, previous rules were designed to predict endpoints occurring within 30 days of ED presentation, which may be an excessively remote perspective, when considering both the viewpoint of the ED and ICU physicians' orientation decisions, and the potential relatedness of a late ICU transfer to physiological alterations caused by pneumonia itself.

Therefore, our goals were to identify risk factors for ICU admission within three days of hospital stay for patients initially presenting without respiratory failure or shock, and to derive and validate a prediction rule to stratify the risk of ICU admission on days 1 to 3.

## Materials and methods

### Study design

This study was based on data obtained from four prospective, multicentre studies in adults with pneumonia. Two were from North America, the Pneumonia Patient Outcomes Research Team (PORT) cohort study and the Emergency Department Community-Acquired Pneumonia (EDCAP) trial, and the two other cohorts were from Europe (Pneumocom-1 and Pneumocom-2). The methods used for the Pneumonia PORT, EDCAP and Pneumocom studies have been reported previously [6-9]. With the exception of the EDCAP cluster randomised trial, all studies were observational. The study protocols were approved by the institutional review boards of the participating institutions. We received permission to use the data from the four original multicentre studies and the need for informed consent for the specific purpose of this study was waived.

### Patients

All studies enrolled consenting adults with pneumonia. Nursing home residents with health care-associated pneumonia were not eligible for the current analysis [10]. Additional exclusion criteria (discharge within 7 to 10 days of presentation, positive HIV antibody titre, immunosuppression, history of cystic fibrosis, ventilation via a tracheostomy or chronic use of mechanical ventilation) varied across the four original studies (Additional data file 1). Patients presenting with acute respiratory failure requiring mechanical ventilation (invasive or noninvasive mechanical ventilation) or shock (systolic arterial pressure below 90 mmHg and requiring vasopressors) who were transferred to the ICU on the same day of ED presentation were considered to have an obvious indication for immediate ICU admission [11] and were excluded from the present analysis. For the purposes of this study, 70% of the patients were randomly assigned to a derivation cohort and 30% to an internal validation cohort.

### Baseline data collection

All four studies used physician interviews and standardised reviews of medical records to collect baseline demographic variables, comorbid illnesses, physical examination findings, laboratory test results and radiographic findings. According to previously published algorithms, prediction rules were derived from each patient's baseline data [6,12,13]. In accordance with methods used in these previous studies, missing variables were assumed to be normal [14,15].

### Outcome measures

**Figure 1:**
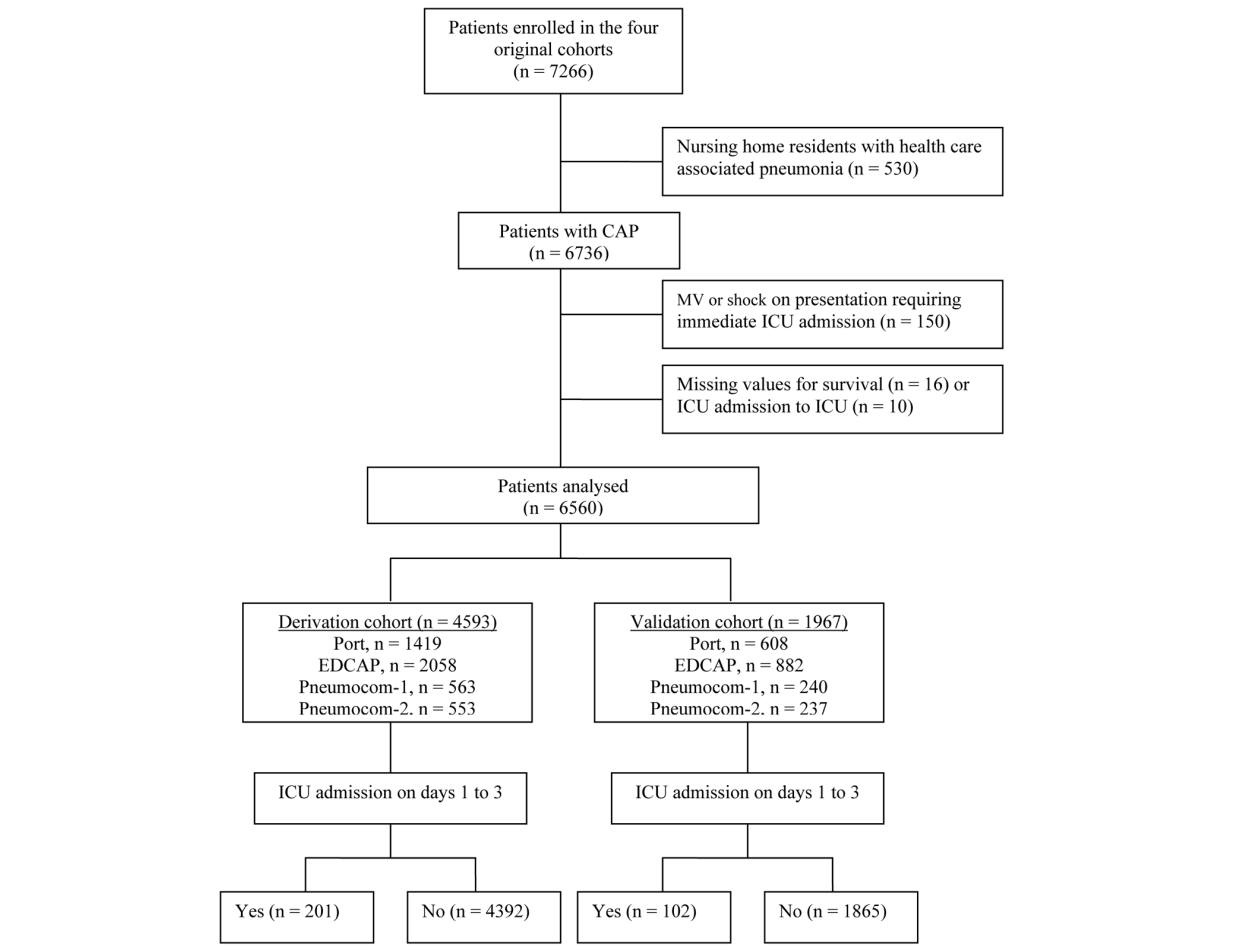
Patient enrolment. CAP = community-acquired pneumonia; EDCAP = Emergency Department Community-Acquired Pneumonia; ICU = intensive care unit; MV = mechanical ventilation.

The primary outcome measure was the occurrence of ICU admission on days 1 to 3 of ED presentation (Figure 1). The secondary outcome was 28-day all-cause mortality.

### Statistical analyses

Baseline and follow-up characteristics were reported as mean and standard deviation or median and interquartile range for continuous variables, and as percentages for discrete variables. We compared patient baseline characteristics according to ICU admission on days 1 to 3, using the two-tailed *t *tests or Wilcoxon tests for continuous variables, and chi-squared tests or the Fisher's exact test for discrete variables.

We first developed a parsimonious logistic regression model by removing variables from the full main effects model using a backward approach with a cut-off value of *P *= 0.10. The variables introduced in the model included demographic characteristics, comorbid conditions and physical, radiographic and laboratory findings. Subsequently, we transformed the regression coefficients of the variables in the final model to an integer value for each variable according to its contribution to the risk estimation. Finally, we derived a four risk class prediction rule for predicting ICU admission on days 1 to 3, and estimated the area under the receiver operating characteristics (ROC) curve for predicting ICU admission on days 1 to 3. We also estimated the area under the ROC curve of our score within each original cohort. All analyses were performed using Stata version 8.0 (Stata Corporation, College Station, TX, USA).

## Results

### Patient characteristics

Overall, 6560 patients were retained in our analysis, including 4593 (70%) in the derivation and 1967 (30%) in the validation cohort (Figure 1). The characteristics of the two cohorts are compared in Tables 1 and 2.

**Table 1:** Patient demographic characteristics, comorbid conditions and baseline physical examination findings

**Characteristics**	**Derivation sample**	**Validation sample**	***P *value**
	(n = 4593)	(n = 1967)	
Demographic factors			
Male gender, n (%)	2428 (52.9)	1040 (52.9)	0.99
Age, mean (SD), years	60 (20)	60 (20)	0.48
Comorbid conditions, n (%)			
Neoplastic disease	229 (5.0)	120 (6.1)	0.06
Liver disease	71 (1.5)	30 (1.5)	0.95
Congestive heart failure	542 (11.8)	235 (11.9)	0.87
Renal disease	243 (5.3)	112 (5.7)	0.51
Coronary artery disease	792 (17.2)	318 (16.2)	0.29
Chronic pulmonary disease	1153 (25.1)	474 (24.1)	0.39
Diabetes mellitus	672 (14.6)	284 (14.4)	0.84
Physical examination findings			
Altered mental status, n (%)	275 (6.0)	120 (6.1)	0.96
Respiratory rate, median (IQR), per minute	22 (20 to 26)	22 (20 to 26)	0.72
Pulse, median (IQR), per minute	97 (84 to 112)	97 (84 to 110)	0.28
Systolic BP, median (IQR), mmHg	132 (118 to 150)	131 (118 to 150)	0.67
Temperature, median (IQR), °C	37.7 (36.8 to 38.5)	37.6 (36.8 to 38.5)	0.07
Oxygen saturation, median (IQR), %	94 (90 to 96)	94 (91 to 97)	0.07

Missing values were assumed to be normal for respiratory rate (n = 819; 12%), pulse (n = 356, 5%), systolic (n = 314, 5%), temperature (n = 323, 5%) and comorbid conditions (< 2%). BP = blood pressure; ICU = intensive care unit; IQR = interquartile range.

**Table 2:** Patient baseline laboratory and x-ray findings, Pneumonia Severity Index and clinical outcomes within 28 days

Characteristics	Derivation sample	Validation sample	*P *value
	(n = 4593)	(n = 1967)	
Laboratory and x-ray findings			
Arterial pH, median (IQR)	7.45 (7.41 to 7.47)	7.44 (7.41 to 7.47)	0.59
Arterial partial pressure of oxygen, median (IQR), mmHg	63 (55 to 74)	64 (55 to 73)	0.62
BUN, median (IQR), mEq/L	6 (4 to 9)	6 (4 to 9)	0.81
Sodium, median (IQR), mEq/L	137 (134 to 139)	137 (134 to 139)	0.63
Glucose, median (IQR), %	7 (6 to 9)	7 (6 to 9)	0.97
Multilobar infiltrates	39 (35 to 42)	39 (36 to 42)	0.48
WBC, median (IQR), G/L	11.7 (8.5 to 15.8)	11.2 (8.1 to 15.3)	0.04
Multilobar infiltrates, n (%)			0.80
Pleural effusion, n (%)	503 (10.9)	206 (10.5)	0.57
Pneumonia Severity Index, n (%)			0.80
Class I	1259 (27.4)	538 (27.3)	
Class II	1075 (23.4)	479 (24.3)	
Class III	877 (19.1)	372 (18.9)	
Class IV	1104 (24.0)	451 (22.9)	
Class V	278 (6.0)	127 (6.5)	
Outcomes			
ICU admission ≤ 3 days, n (%)	201 (4.4)	102 (5.2)	0.15
3-day mortality, n (%)	41 (0.9)	12 (0.6)	0.24
28-day ICU admissions, n (%)	259 (5.6)	119 (6.0)	0.51
28-day mortality, n (%)	184 (4.0)	78 (4.0)	0.94

Missing values were assumed to be normal for arterial pH (n = 4247, 65%), arterial partial pressure of oxygen or oxygen saturation (n = 1029, 15%), BUN (n = 1685, 26%), sodium (n = 1565, 24%), glucose (n = 1637, 25%), haematocrit (n = 1205, 18%), WBC (n = 1185, 18%). BP = blood pressure; BUN = blood urea nitrogen; ICU = intensive care unit; IQR = interquartile range; WBC = white blood cell.

### Outcomes measures

During the 28-day follow-up, 378 patients were admitted to an ICU (5.6% and 6.0%, respectively in the derivation and validation cohorts; Table 2). More than 80% of ICU admissions occurred within three days of ED presentation. Conversely, nearly 80% of the 262 deaths occurred after three days, whereas about 20% (53) of the deaths occurred within three days of presentation.

### Factors associated with ICU admission on days 1 to 3

#### Baseline characteristics associated with ICU admission on days 1 to 3

Patients admitted to the ICU on days 1 to 3 were more likely to be elderly men with comorbidities, and to have more vital sign abnormalities (altered mental status, tachypnoea and hypotension), radiographic or laboratory abnormalities (hypoxaemia, hyponatraemia, acidosis, high blood urea nitrogen level, and pleural effusion or multilobar infiltrates; Tables 3 and 4).

**Table 3:** Association of patient demographic characteristics, comorbid conditions and baseline physical examination findings with intensive care unit admission within three days of presentation

**Characteristics**	**Derivation cohort**			**Validation cohort**		
	Admission to ICU ≤ 3 days		*P *value	Admission to ICU ≤ 3 days		*P *value*
				
	**No (4392)**	**Yes (201)**		**No (1865)**	**Yes (102)**	
**Demographic factors**						
Male gender, %	52.4	63.7	0.002	52.5	59.8	0.15
Age, mean (SD) years	59 (21)	67 (15)	< 0.001	60 (21)	65 (16)	0.01
**Comorbid conditions, %**						
Cancer	5.0	5.5	0.75	5.8	11.8	0.01
Cerebrovascular disease	6.8	8.9	0.15	6.9	6.5	0.87
Liver disease	1.5	20.	0.60	1.4	3.9	0.04
Congestive heart failure	11.2	25.9	< 0.001	11.4	21.6	0.002
Renal disease	4.9	14.4	< 0.001	5.4	10.8	0.02
Coronary artery disease	16.6	30.8	< 0.001	15.9	20.6	0.21
Chronic pulmonary disease	24.8	31.3	0.04	23.9	28.4	0.29
Diabetes mellitus	14.3	21.4	0.006	13.7	27.4	< 0.001
**Physical examination findings, %**						
Altered mental status	5.5	15.9	< 0.001	5.7	12.7	0.004
Respiratory rate ≥ 30 breaths/minute	12.3	33.8	< 0.001	11.5	35.3	< 0.001
Systolic BP < 90 mmHg	1.5	5.0	< 0.001	1.2	2.0	0.48
Temperature < 35 or ≥ 40°C	5.6	9.4	0.02	5.8	12.7	0.005
Pulse ≥ 125 beats/minute	8.8	18.4	< 0.001	7.5	23.5	< 0.001
Oxygen saturation, < 90%	13.6	44.4	< 0.001	14.0	46.4	< 0.001

Admission to ICU ≤ 3 days refers to patients who were admitted to an ICU within 3 days of presentation at the emergency department. * *P *value refers to the variables associated with admission to ICU within 3 days of presentation.

BP = blood pressure; ICU = intensive care unit; SD = standard deviation.

**Table 4:** Association of patient laboratory and x-ray findings, and Pneumonia Severity Index with ICU admission within three days of presentation

**Characteristics**	**Derivation cohort**			**Validation cohort**		
	Admission to ICU ≤ 3 days		*P *value	Admission to ICU ≤ 3 days		*P *value*
				
	**No (4392)**	**Yes (201)**		**No (1865)**	**Yes (102)**	
**Laboratory and x-ray findings, %**						
Arterial pH < 7.35	2.3	10.9	< 0.001	2.6	19.6	< 0.001
BUN ≥ 10 mmol/L	13.0	37.8	< 0.001	12.9	30.4	< 0.001
Sodium < 130 mEq/L	3.9	11.7	< 0.001	3.0	13.0	< 0.001
Glucose ≥ 14 mmol/dL	4.8	9.9	0.001	5.1	10.9	0.02
Haematocrit < 30%	4.5	10.9	< 0.001	4.7	11.8	0.002
WBC < 3 or ≥ 20 G/L	9.1	18.9	< 0.001	8.4	18.6	< 0.001
PaO_2 _< 60 mmHg	21.9	54.7	< 0.001	18.9	56.9	< 0.001
Pleural effusion	10.4	21.9	< 0.001	10.1	17.6	0.01
Multilobar infiltrates	22.0	41.8	< 0.001	22.2	39.2	< 0.001
**Pneumonia Severity Index, %**						
Class I	28.4	5.0	< 0.001	28.6	4.9	< 0.001
Class II	24.0	10.9		24.9	13.7	
Class III	19.1	17.9		18.7	22.5	
Class IV	23.0	46.8		22.2	35.3	
Class V	5.4	19.4		5.5	23.5	

Admission to ICU ≤ 3 days refers to patients who were admitted to an ICU within three days of presentation to the emergency department. * *P *value refers to the variables associated with admission to ICU within 3 days of presentation. BUN = blood urea nitrogen; ICU = intensive care unit; PaO_2 _= arterial partial pressure of oxygen; WBC = white blood cell.

#### Independent risk factors

In multivariable analysis, we identified 11 independent predictors of ICU admission on days 1 to 3, including male gender, age under 80 years and at least one comorbid condition; all other independent risk factors were physical or laboratory findings (Table 5).

**Table 5:** Adjusted coefficients and odd ratios for admission to ICU within three days of presentation and points assigned in the predictive model

**Characteristics**	**β** **parameter**	**95% CI (β parameter)**	**OR**	**95% CI (OR)**	**Points** **assigned**
Male	0.39	(0.08 to 0.70)	1.47	(1.08 to 2.01)	1
Comorbid condition ≥ 1	0.45	(0.11 to 0.78)	1.57	(1.12 to 2.19)	1
Respiratory rate ≥ 30 breaths/minutes	0.53	(0.18 to 0.88)	1.70	(1.20 to 2.41)	1
White blood cell count < 3 or ≥ 20 G/L	0.54	(0.14 to 0.94)	1.71	(1.15 to 2.55)	1
Heart rate ≥ 125 beats/minute	0.55	(0.14 to 0.95)	1.73	(1.15 to 2.60)	1
Age < 80 years	0.57	(0.18 to 0.95)	1.76	(1.19 to 2.59)	1
Multilobar infiltrates or pleural effusion	0.79	(0.48 to 1.09)	2.19	(1.62 to 2.97)	2
Oxygen saturation< 90% or PaO_2 _< 60 mmHg	0.85	(0.53 to 1.17)	2.35	(1.71 to 3.23)	2
Arterial pH < 7.35	0.91	(0.38 to 1.44)	2.49	(1.47 to 4.22)	2
Blood urea nitrogen ≥ 11 mmol/L	0.94	(0.61 to 1.28)	2.56	(1.84 to 3.58)	2
Sodium < 130 mEq/L	1.06	(0.58 to 1.53)	2.88	(1.79 to 4.63)	3

CI = confidence Interval; OR = odds ratio; PaO_2 _= arterial partial pressure of oxygen.

#### Risk of early admission to the ICU

The risk of early admission to the ICU (REA-ICU) score ranged from 0 to 17 and was stratified into four risk classes (REA-ICU index; Table 6). In the derivation cohort the rate of ICU admission on days 1 to 3 ranged from 1.1% for risk class I to 27.1% for risk class IV and 28-day mortality ranged from 1.2 to 15.1%. Similar rates were observed in the validation cohort. In risk class I, five patients (not admitted to ICU) died within three days of ED presentation. The risk class I patients accounted for 2510 of 4593 (54.6%) and 1099 of 1967 (55.9%) patients, respectively, in the derivation and validation cohorts, with 27 out of 2510 (1.1%) and 14 out of 1099 (1.3%) of these patients admitted to the ICU, respectively. Among these 41 patients, 10 were classified as high-risk using the Pneumonia Severity Index (PSI) and none subsequently died.

**Table 6:** Population and outcomes stratification according to the risk of early ICU admission index (REA-ICU index) of patients with community acquired pneumonia

		**Derivation population**			**Validation population**		
			
**Risk class**	**Score**	N	ICU ≤ 3 days, % (95% CI)	Death ≤ 28 days, % (95% CI)	n	ICU ≤ 3 days, % (95% CI)	Death ≤ 28 days, % (95% CI)
I	≤ 3	2510	1.1 (0.7 to 1.6)	1.2 (0.8 to 1.8)	1099	1.3 (0.7 to 2.1)	1.9 (1.2 to 2.9)
II	4 to 6	1498	5.5 (4.4 to 6.8)	6.0 (4.8 to 7.3)	633	7.1 (5.2 to 9.4)	4.4 (3.0 to 6.3)
III	7 to 8	419	11.0 (8.2 to 14.4)	9.1 (6.5 to 12.2)	164	12.2 (7.6 to 18.2)	7.9 (4.2 to 13.2)
IV	≥ 9	166	27.1 (20.5 to 34.5)	15.1 (10.0 to 21.4)	71	32.4 (21.7 to 44.5)	22.5 (13.5 to 34.0)

Total		4593	4.4 (6.0 to 7.4)	4.0 (3.4 to 4.6)	1967	5.2 (5.8 to 8.0)	4.0 (3.1 to 4.9)

ICU ≤ 3 days and death ≤ 28 days refer to patients who were admitted to an ICU within three days of presentation to the emergency department or who died within 28 days of presentation, respectively. Results are expressed as percentages of each outcome within each REA-ICU risk class. CI = confidence interval; ICU = intensive care unit.

The area under the ROC curves for the REA-ICU score was 0.80 (95% confidence interval (CI) = 0.77 to 0.83) and 0.80 (95% CI = 0.76 to 0.84) in the derivation and validation cohorts, respectively.

The risk of admission to the ICU on days 1 to 3 increased significantly from risk class I to risk class IV within each of the four original cohorts (*P *< 0.001 for each cohort). The area under the ROC curve of the score for predicting admission to an ICU on days 1 to 3 ranged from 0.76 (95% CI = 0.72 to 0.90) in the EDCAP cohort to 0.82 (95% CI = 0.85 to 0.90) in the Pneumocom-2 cohort.

The REA-ICU score yielded a higher area under the ROC curve than the PSI (0.75, 95% CI = 0.73 to 0.78), CURB-65 (0.69, 95% CI = 0.66 to 0.72) and Espana Severe CAP (SCAP) (0.74, 95% CI = 0.71 to 0.76) for predicting ICU admission on days 1 to 3 for patients not requiring immediate circulatory or ventilatory support (*P *< 0.001 for all pairwise comparisons involving the REA-ICU score).

## Discussion

In this study, we identified 11 baseline characteristics that were independently associated with ICU admission on days 1 to 3 in a broad range of patients presenting with CAP and no obvious reason for immediate ICU admission (i.e. not requiring immediate respiratory or circulatory support). These characteristics included male gender, age younger than 80 years, comorbid condition of 1 or higher, tachypnoea, tachycardia, leukopenia or leukocytosis, multilobar infiltrates or pleural effusion, hypoxaemia, acidosis, hyperuraemia and hyponatraemia. From this set of variables, we derived a prediction rule, REA-ICU score, that demonstrated a consistent discriminatory power for predicting ICU admission occurring within three days of ED presentation for patients with CAP not requiring immediate ICU transfer.

The British Thoracic Society advocates using a set of only four variables (CURB-65) and suggests considering ICU referral when three or more criteria are present [13]. The ATS rule, modified in 2001 [16], appears to have a slightly better predicting accuracy than the CURB-65 or the PSI; however, it still results in a substantial proportion of patients misclassified with regard to ICU admission [17]. Moreover, the two major criteria of the ATS rule – requirements for mechanical ventilation and the occurrence of shock – are obvious reasons for ICU admission. Espana and colleagues derived the SCAP prediction rule that was shown to discriminate better than previous prediction rules between ED patients with and without CAP-related adverse medical outcomes, including 30-day mortality and ICU referral [12]. Narrowing the criteria for severe CAP needing ICU admission to the requirement for intensive respiratory or vasopressor support (IRVS), Charles and colleagues recently developed the SMART-COP, which demonstrated interesting characteristics to predict IRVS requirement during the whole hospital course of patients [18]. We took a different perspective and focused on patients not presenting to the ED with a need for IRVS, but subsequently transferred to the ICU within the first three days of admission; thus, our index might be especially useful for emergency physicians to assess the potential risk of ICU requirement within the next few days among those patients presenting with none of the ATS major severity criteria. As a result, the REA-ICU performed significantly better than existing prediction rules (PSI, CURB-65, Espana SCAP) in predicting ICU admission on days 1 to 3 of ED presentation in these patients.

Indeed, the criteria for inclusion in our analysis have several distinctive features from previous attempts at predicting CAP severity. First, contrasting with previous prediction rules, we focused on the more challenging subgroup of patients presenting with moderately severe CAP and no requirement for immediate ICU admission [11]; hence, we excluded patients with obvious respiratory or haemodynamic failure at presentation. Indeed, including such clinically apparent features in a prediction rule is likely to improve its operative characteristics, but is of limited value in assisting physicians in triaging patients [19,20].

Second, we focused on admission to ICU within three days of ED presentation, instead of including all 28-day outcomes. Pneumonia is the most common cause of severe sepsis, and severe CAP should be seized in the overall context of sepsis from pulmonary infection with organ dysfunction(s) potentially requiring intensive care [5,21]. Indeed, most sepsis-related organ failures in this setting occur early [3,22]. Accordingly, our findings in a large sample of patients presenting with CAP confirm that admission to ICU mostly occurred within the first three days of ED presentation. In addition, late ICU admissions may be associated with other factors than the severity of pneumonia itself (e.g. decompensated comorbidity or an intercurrent event), and not be influenced by its initial management [23-25]. Moreover, the REA-ICU score was based on data readily available at patient presentation to the ED and did not include results from ED monitoring, which would be less relevant to triaging patients in the ED setting [12,26]. Accordingly, we could not include laboratory tests that were not evenly collected across the four original studies (e.g. albuminaemia).

Third, we considered that adequate ICU admission should not be restricted to patients requiring IRVS [19]. Indeed, ICU care has been demonstrated to improve outcome in severely ill and unstable patients, and these patients require intensive monitoring and may potentially need immediate intervention [27]. Therefore, given the characteristics of the REA-ICU (Additional data file 2), we suggest that intensive care physicians be informed of those patients with the highest risk of three-day ICU admission. This could be achieved by requesting the advice of an intensivist for such patients, who would then help decide on the most appropriate site of care for providing them adequate management and close monitoring, possibly in the ICU or an intermediate-care unit as deemed appropriate.

Fourth, despite substantial differences across the four original cohorts in patient characteristics and outcomes (Tables 1 and 2) [6-9], the overall discriminatory power of the REA-ICU score in predicting ICU admission on days 1 to 3 was quite high across the four original cohorts, reflecting the robustness of this score [28].

Several potential limitations of our study must be acknowledged. First, there were slight methodological differences and exclusion criteria across the four cohorts analysed. However, the definitions used in EDCAP, Pneumocom-1 and Pneumocom-2 were all based on the Pneumonia PORT study. Second, our findings do not take into account processes of care or causative pathogens, which may have confounded the relation between risk class and patient outcomes. As these data were not collected in a standardised manner across the four studies, we could not adjust for these variables. Third, the REA-ICU score includes 11 variables, which might limit its applicability to clinical use. However, the 20-variable PSI has been successfully implemented in various settings, including routine practice [7,9,29-31]. Fourth, our findings are based solely on hospital admission data and patient monitoring data were not recorded during the initial hospital course, so we could not analyse the adequacy of secondary ICU admission (e.g. requirement for mechanical ventilation or vasopressor, or other reason for ICU admission). Fifth, all laboratory tests were performed at the discretion of the attending physicians and missing values were assumed to be normal. This strategy is widely used in the clinical application of prediction rules and reflects the methods used in the original derivation and validation of the PSI [15]. Indeed, patients with less severe illness were more likely to have missing values for laboratory findings. Finally, prediction scores often perform better in their derivation and internal validation cohorts than in external validation studies; therefore, external independent validation is required.

## Conclusions

In summary, using a large database combining four prospective cohorts of patients with CAP, we derived and validated the REA-ICU index to predict ICU referral within the first three days of hospital admission in patients without overt circulatory or respiratory failure at ED presentation. This index demonstrates valuable characteristics for stratifying the risk of admission to ICU on hospital days 1 to 3. Using this combination of variables might help ED physicians to more accurately assess the potential need for ICU admission in the challenging group of high-risk patients presenting with no obvious reason for ICU admission [5,32,33].

## Key messages

• Among 6560 patients with CAP and no obvious indication for ICU admission at ED presentation, 303 (4.6%) were admitted to the ICU within the three following days.

• Eleven variables – male gender, older age, comorbid conditions, tachypnoea, tachycardia, multilobar infiltrate or pleural effusion, low or high white blood cell count, hypoxaemia, high blood urea nitrogen, acidosis, hyponatraemia – were independently associated with admission to ICU on days 1 to 3, and were used to derivate the REA-ICU index.

• The REA-ICU index stratified ED patients with CAP and no obvious indication for ICU admission into four classes of risk for ICU admission on days 1 to 3, ranging from 0.7 to 31%. This index might help ED physicians and intensivists in the disposition decision.

## Abbreviations

ATS: American Thoracic Society; CAP: community-acquired pneumonia; CI: confidence interval; ED: emergency department; EDCAP: Emergency Department Community-Acquired Pneumonia; ICU: intensive care unit; IRVS: intensive respiratory or vasopressor support; OR: odds ratio; PORT: Patient Outcomes Research Team; PSI: Pneumonia Severity Index; REA-ICU: risk of early admission to ICU; ROC: receiver operating characteristics; SCAP: severe community-acquired pneumonia.

## Competing interests

MJF consults for the University of Pennsylvania and GeneSoft Pharmaceuticals Inc. He also receiveds honoraria from Zynx Health Corporation, STA Healthcare Communications Inc., University of Alberta and Maine Medical Center). MJF gives expert testimony for Stephen Lynn Klein, Kellogg & Siegelman, Swanson, Martin, & Bell, William J. Burke, Chad McGowan, Chernett, Wasserman, Yarger and Pasternak, LLC. MJF received grants from Pfizer Inc. BR received grants from GlaxoSmithKline Inc. MJF also received royalties from Up-to-Date.

## Authors' contributions

BR, JL, CBB made substantial contributions to conception and design. BR, JL, EC, AS, MG, NC, ER, FH, JH, MS, MJF and CBB made substantial contributions to acquisition of data. BR, JL, EC, AS, NC, MS, MJF and CBB made substantial contributions to analysis and interpretation of data. BR, JL, EC, AS, MG, MJF, FH, JH and CBB were involved in drafting the manuscript or revising it critically for important intellectual content. BR, JL, EC, AS, MG, NC, ER, FH, JH, MS, MJF and CBB gave their final approval of the version to be published. BR, EC, AS, MG, ER, JH, MS and MJF were involved in acquisition of funding and collection of data. BR, EC, AS, MG, MJF and CBB were involved in general supervision of the research group.

## Supplementary Material

Additional file 1 Word file containing a table comparing study patient exclusion criteria across the four original study populations. Click here for file

Additional file 2 Word file containing a table that describes the risk of early intensive care unit admission index characteristics. Click here for file
