# Supplementary material for: Risk stratification of early admission to the intensive care unit of patients with no major criteria of severe community-acquired pneumonia: development of an international prediction rule
Source: Crit Care. 2009 Apr 9;13(2):R54. doi: 10.1186/cc7781 (PMC2689501; doi:10.1186/cc7781)
Supplement: Additional file 1 — Word file containing a table comparing study patient exclusion criteria across the four original study populations. [file cc7781-S1.doc]

**Table S1**. Comparison of Study Patient Exclusion Criteria Across Study Populations

| **Exclusion criteria** | **Pneumonia**  **PORT** | **EDCAP** | | **Pneumocom-1** | | **Pneumocom-2** | |
| --- | --- | --- | --- | --- | --- | --- | --- |
|  |  |  |  | |  |  |  |
| Discharge within 7–10 days of presentation* | X | X | X | |  | X |  |
| Positive HIV antibody titer | X | X | X | |  | X |  |
| Immunosuppression^†^ | -- | X | X | |  | X |  |
| History of cystic fibrosis | -- | X | X | |  | -- |  |
| Ventilated via a tracheotomy or chronic use  of mechanical ventilation | -- | X | X | |  | -- |  |
| Other^‡^ | -- | X | -- | |  | -- |  |

* Seven days for the Pneumocom-1 and Pneumocom-2 studies and 10 days for the Pneumonia PORT and EDCAP studies.

^†^ Immunosuppression was defined as (1) active cancer, leukemia, or lymphoma; (2) white blood cell count <3000/mm^3^ or an absolute neutrophil count <1000/mm^3^ on presentation; (3) asplenia, anatomic or functional; (4) hypogammaglobulinemia; (5) immunosuppressive or myelosuppressive drug therapy within the 30 days preceding presentation; (6) documentation of patient “on chemotherapy,” or (7) radiation therapy.

^‡^ Other exclusion criteria consisted of: (1) pulmonary tuberculosis; (2) confirmed diagnosis of pneumonia within 30 days of presentation; (3) transfer from an acute care hospital or on-site nursing care facility; (4) residence in a chronic care hospital immediately before presentation; (5) psychosocial problems incompatible with outpatient treatment, enrollment, or follow-up; (6) pregnancy; (7) illicit drug use within the past 30 days; (8) alcoholism with evidence of end-stage organ damage; (9) homelessness; (10) incarceration as a prisoner; (11) admission for palliative care; or (12) previous enrollment in a competing research protocol.
