# Supplementary material for: Risk stratification of early admission to the intensive care unit of patients with no major criteria of severe community-acquired pneumonia: development of an international prediction rule
Source: Crit Care. 2009 Apr 9;13(2):R54. doi: 10.1186/cc7781 (PMC2689501; doi:10.1186/cc7781)
Supplement: Additional file 2 — Word file containing a table that describes the risk of early intensive care unit admission index characteristics. [file cc7781-S2.doc]

**Table S2 Characteristics of the REA-ICU Index**

| Cut-off | Se* | Sp* | PPV* | NPV* | LR+* | LR-* |
| --- | --- | --- | --- | --- | --- | --- |
| 2 ≤ | 86.5  (85.6–87.3) | 57.0  (55.8–58.2) | 8.9  (8.2–9.6) | 98.9  (98.6–99.1) | 2.0  (1.9–2.1) | 0.23  (0.19–0.31) |
| 3 ≤ | 44.2  (43.0–45.4) | 89.0  (88.3–89.8) | 16.3  (15.4–17.2) | 97.1  (96.6–97.5) | 4.0  (3.5–4.7) | 0.63  (0.57–0.69) |
| 4 ≤ | 22.4  (21.4–23.4) | 97.3  (96.9–97.7) | 28.7  (27.6–29.8) | 96.3  (95.8–96.7) | 8.3  (6.4–10.7) | 0.80  (0.75–0.85) |

*Results are given as estimates and 95% confidence intervals.

Abbreviations: REA-ICU: Risk of Early Admission to Intensive Care Unit; Se: Sensitivity; Sp: Specificity, PPV: Positive Predictive Value; NPV: Negative Predictive Value; LR+: Likelihood Ratio Positive; LR-: Likelihood Ratio Negative
